# Performance Evaluation, Thermodynamic Analysis and Cost Assessment of a Stand-Alone Desalination Plant Driven with PV-Solar Without Battery Support

**DOI:** 10.3390/membranes16050176

**Published:** 2026-05-15

**Authors:** Manuela Celeste Salgado-Pineda, Jonathan Ibarra-Bahena, Yuridiana Rocio Galindo-Luna, Eduardo Venegas-Reyes, José Agustín Breña-Naranjo, Ulises Dehesa-Carrasco

**Affiliations:** 1 Posgrado en Ciencias y Tecnología del Agua, Instituto Mexicano de Tecnología del Agua, Paseo Cuauhnáhuac 8532, Jiutepec 62550, Mexico; manuela.salgado@posgrado.imta.edu.mx; 2 Instituto de Energías Renovables, Universidad Nacional Autónoma de México, Privada Xochicalco S/N, Temixco 62580, Mexico; jibarra@ier.unam.mx; 3 Departamento de Ingeniería de Procesos e Hidráulica, Universidad Autónoma Metropolitana-Iztapalapa, Av. Ferrocarril San Rafael Atlixco 186, Col. Leyes de Reforma 1 A sección, Iztapalapa, Ciudad de México 09310, Mexico; ygalindol@izt.uam.mx; 4 Subcoordinación de Agua, Energía y Proyectos Productivos, Instituto Mexicano de Tecnología del Agua, Paseo Cuauhnáhuac 8532, Jiutepec 62550, Mexico; eduardo_venegas@tlaloc.imta.mx; 5 Instituto de Ingeniería, Universidad Nacional Autónoma de México, Cd. Universitaria, Coyoacán, Ciudad de México 04510, Mexico; agustin.brena@ii.unam.mx; 6 El Colegio de Chihuahua, Partido Díaz 4723, Cd. Juárez 32310, Mexico

**Keywords:** brackish water, desalination, exergy analysis, cost assessment

## Abstract

Desalination by reverse osmosis (RO) of brackish water and seawater is a cost-competitive solution for supplying irrigation water in off-grid and water-stressed regions. This paper presents an experimental evaluation, thermodynamic analysis, and cost assessment of a solar photovoltaic brackish-water reverse osmosis (PV-BWRO) desalination system. Five feed salinity levels ranging from 993.6 to 3191.8 mg/L were tested. The results show that water production decreased with increasing feed salinity, from 3.29 m^3^/day at 24.6 mg/L to 1.48 m^3^/day at 152.9 mg/L. The calculated specific energy consumption values ranged from 1.80 to 3.61 kWh/m^3^ for solar irradiances of 1005.99 and 1018.47 W/m^2^, respectively. An exergy analysis revealed that the solar panels and pump operated at efficiencies of 11.7% and 38.9%, while exergy destruction was mainly concentrated in the pretreatment stage (28.72%), membrane modules (42.5%), and reject stream (28.5%). Although the overall system efficiency remained low (maximum of 1.39%), the results highlight substantial potential for improvement through enhanced maintenance, optimized pretreatment, and exergy recovery strategies. The unit water production cost ranged from USD 0.49 at 993.6 mg/L to USD 1.87 at 3191.8 mg/L, assuming a target permeate total dissolved solids concentration of 500 mg/L.

## 1. Introduction

As the global population and industrial water demand increase, the availability of freshwater resources is becoming a strategic issue for countries and regions. One way to address the freshwater crisis is through the desalination of brackish water sources. Brackish water sources are mainly groundwaters, it was estimate 22.6 million km^3^ of groundwater which probably are saline of brackish [1]. The salinity range of brackish sources is 1000 to 10,000 mg/L of total dissolved solids (TDS), and they typically contain low levels of organic carbon as well as colloidal contaminants [2].

Among desalination technologies, membrane-based processes are the most widely used because they provide several advantages: high processing capacity, adaptability to source water quality, compact equipment size, simple operation, and low capital and operating costs [3,4]. Brackish water desalination by reverse osmosis (BWRO) consumes less energy than seawater desalination, which means that the use of renewable energies is feasible. In particular, PV-powered BWRO systems can operate off-grid, using solar energy directly, which makes them suitable for remote or rural areas where no electrical infrastructure is available [5]. This reduces environmental impacts, making it a suitable alternative for domestic water supply or irrigation [6,7]. This concept has been widely reported in the literature: Bdour et al. [8] evaluated the performance of a BWRO plant at the Hashemite University in Jordan that was powered by a photovoltaic (PV) system. According to the authors, the specific energy consumption (SEC) was 1.2 kWh/m^3^, which is 140% to 400% lower than that of other desalination plants of similar capacity. The operational cost of this plant was calculated at USD 0.23/m^3^, which is 260% lower than that of local grid-powered desalination plants. Hekmatmehr et al. [9] designed a 350 m^3^/day BWRO system based on local water-quality data collected over a 9-year period. A PV system without battery support was sized to power the desalination plant. The authors found that increasing the TDS in the feed water from 1313 to 6938 mg/L resulted in a 0.45 kWh rise in SEC, while raising the temperature from 12 to 36 °C reduced the SEC by 0.2 kWh. Tigrine et al. [10] carried out a feasibility study of a PV-BWRO plant with a daily production of 2 m^3^ of freshwater in Algeria. According to the authors, brackish water with a TDS of 5 g/L requires 1.5 kWh/m^3^ for the desalination process, whereas for seawater with a TDS of 35 g/L, this requirement increases to 5.6 kWh/m^3^. Cervantes-Rendón et al. [11] evaluated the performance of a PV-BWRO plant operating without battery support. The system produced up to 1.8 m^3^/day of freshwater from a feed salinity of 2921 mg/L, and the estimated freshwater cost was USD 2.22/m^3^. Dawoud et al. [12] analyzed a PV-BWRO system without battery support to produce 1000 m^3^/day from a feed salinity of 25,000 ppm. The authors estimated the production cost to range from USD 0.55 to 0.63/m^3^ and concluded that the proposed system is an environmentally friendly and a cost-efficient solution for mitigating water scarcity. Extensive literature reviews on PV-BWRO systems were carried out by Al-Obaidi et al. [13] and Elfaqih et al. [14].

Regarding agricultural applications, desalination of brackish water with TDS levels from 3000 to 10,000 mg/L has great potential to support the sustainability of irrigated agriculture and to reduce the impacts of climate change in water-scarce regions [15]. Rezk et al. [16] analyzed a PV-BWRO system with battery support designed to produce 350–500 m^3^/day in summer and 200–250 m^3^/day during colder periods, using a feed stream with a salinity of 2500 mg/L for irrigation in Al Minya (Egypt). According to the authors, the optimal configuration of the system included a 75 kW PV array, a battery bank consisting of 32 Trojan L16P units, and a 28 kW converter, with a total net present cost (NPC) of USD 109,856 and an energy cost of USD 0.059/kWh. Dehesa-Carrasco et al. [17] evaluated the performance of a low-pressure desalination system without battery support for agricultural applications in rural areas. The PV-BWRO system consisted of eight polycrystalline silicon modules providing a nominal power of 1.92 kW, a SQFlex 16 SQF-10 direct-current submersible centrifugal pump, and four ESNA1-LF-4040 polyamide membranes. According to the authors, the system produced 2.16–4.8 m^3^/day of freshwater at a unit cost of USD 1.05–0.47/m^3^, with a maximum energy consumption of 1.55 kWh/m^3^ for a feedwater concentration of 2539 mg/L. Atab et al. [18] carried out a numerical analysis to evaluate the performance of a BWRO system for potable and irrigation water production located at the Main Outfall Drain in Iraq. The authors reported that, the total water cost of a BWRO plant with a capacity of 24,000 m^3^/day, using a feed stream with a salinity of 15,000 ppm (to produce freshwater with <400 ppm), was GBP £0.11/m^3^, and the investment cost was calculated as GBP £14.4 million. The cost of production for irrigation, with the same production volume and feed salinity (to produce freshwater with <1600 ppm), was GBP £0.9/m^3^, with an investment cost of GBP £11.3 million.

Although the specific energy consumption (SEC) is widely accepted as a quantitative metric for desalination processes, exergy analysis not only identifies the components with the greatest exergy destruction but also evaluates the performance of the entire system through the second-law, or exergetic, efficiency. Higher efficiency indicates that the system operates closer to an ideal reversible process [19,20].

This paper presents the performance of a stand-alone solar-PV driven desalination plant without battery support for irrigation purposes, including a first and second-law thermodynamic analysis and an economic assessment.

## 2. Materials and Methods

### 2.1. PV-BWRO System

The experimental PV-BWRO system was integrated by six solar polycrystalline silicon modules JKM270PP 60 (Jinko Solar Co., Ltd., Shanghai, China), which provide a nominal power of 1.620 kW; a 16 SQF 10 submersible pump (by Grundfos, Bjerringbro, Denmark); and four RO low pressure desalination modules with RE4040-BLN polyamide membranes (by Toray Advanced Materials Korea Inc., Jeollanam-do, Republic of Korea). To measure the influent and permeate volumetric flows, two water flow sensors (Steren, Ciudad de México, Mexico) were used, and the reject flow was calculated using mass conservation. Three G2 pressure transducers (Ashcroft Inc., Stratford, CT, USA) were employed to measure the pressure of each stream. T-type thermocouples (Omega Engineering, Norwalk, CT, USA) were used to measure the stream temperatures. A SP Lite2 pyranometer (Kipp & Zonen, Delft, The Netherlands) was used to measure the solar radiation. A multiparameter sensor device (Hanna Instruments, Smithfield, RI, USA) was used to measure the salinity of the streams, for this, Total Dissolved Solids (TDS) unit was used. An Agilent data acquisition unit (Agilent Technologies, Santa Clara, CA, USA) recorded the experimental data. The uncertainties of the measured variables and instruments used in the experimental test runs are shown in Table 1. A pretreatment filter was used to remove particles higher than 5 μm.

The operation of the system is as follows: Solar panels convert solar radiation into electrical energy to operate the pump. The pump extracts brackish water from the influent and increases its pressure. The pressurized stream then enters the pretreatment stage, where suspended solids down to 5 microns are removed. The resulting stream is subsequently fed to low-pressure RO membranes, where the desalination process takes place. The permeate is produced as the product stream, while the brine corresponds to the rejected stream. To maintain constant chemical operating conditions during the experiment, both the reject stream and the permeate are returned to the influent after being measured.

**Figure 1:**
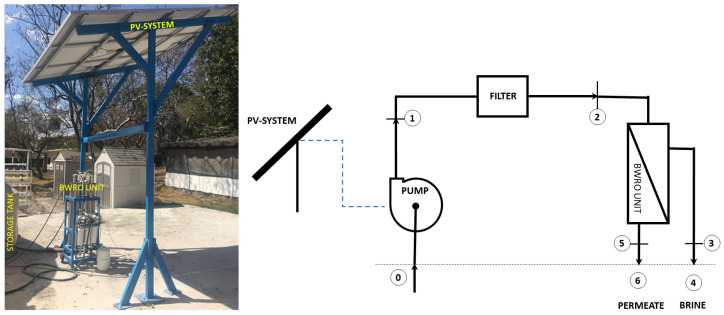
Experimental PV-BWRO system.

The experimental tests were conducted in Jiutepec, Morelos, Mexico (18°53′06.21″ N, 99°09′31.86″ W). Synthetic water samples were prepared with total dissolved solids (TDS) concentrations ranging from 993.6 ± 21.2 mg/L to 3191.8 ± 64.8 mg/L. The experimental operating conditions are shown in Table 2. Figure 1 shows the experimental system and a schematic diagram of the setup.

### 2.2. Thermodynamic Analysis

To evaluate the performance of the experimental PV-BWRO system, first- and second-law thermodynamic analyses were performed. The specific energy consumption (*SEC*) represents the relationship between the energy supplied by the solar PV system and the volume of permeate produced:

(1) $$SEC=\frac{W_{PV}}{{\dot{m}}_{p}}$$

where *ṁ_p_* denotes the average permeate flow over time as a function of solar radiation, and *W_PV_* denotes the average electric power supplied to the pumping system during the same period.

A first-law analysis provides the specific energy efficiency of the system. In contrast, exergy analysis incorporates both the first and second laws of thermodynamics. It identifies the components that generate the highest entropy, which reduces overall system effectiveness. Consequently, it reveals where the major exergy losses occur.

The exergy of a system is defined as the maximum useful work that can be produced from the combination of the system and a specified reference environment, which is assumed to be infinite and in equilibrium. Exergy is destroyed when an irreversible process occurs, [21]. Exergy balance for any system and process can be expressed as:

(2) $${Ex}_{in}-{Ex}_{out}-{Ex}_{dest}=∆Ex$$

where *Ex_in_* is the total exergy entering the system, *Ex_out_* is the total exergy leaving it, *Ex_dest_* is the total exergy destroyed during the process, and Δ*Ex* represents the change in total exergy.

The exergy flow associated with a given stream (*Ψ*) is calculated as follows:

(3) $$\Psi =h-h_{0}-T_{0}\left(s-s_{0}\right)$$

where *T*_0_ is the dead-state temperature (K), and *h*_0_ and *s*_0_ are the specific enthalpy (kJ/kg) and specific entropy (kJ/kg·K) at the dead state, respectively.

The rate of exergy transfer associated with a fluid stream (*ṁ*) can be expressed as:

(4) $$Ex=\Psi \cdot \dot{m}=\dot{m}\left[h-h_{0}-T_{0}\left(s-s_{0}\right)\right]$$

The enthalpy and entropy of the inlet and outlet streams with different salt concentrations were calculated using the correlation reported by Banat and Jwaied [22]:

(5) $$h=w_{s}h_{s}+w_{w}h_{w}$$

(6) $$s=w_{s}s_{s}+w_{w}s_{w}$$

where *w* is the weight fraction, and the subscripts *s* and *w* represent salt and water, respectively.

The enthalpy and entropy values of pure water were estimated using IAPWS-IF97 tables. Whereas the corresponding values for the salt were calculated as follows:

(7) $$h_{s}=h_{s_{0}}+{Cp}_{s}\left({T-T}_{0}\right)$$

(8) $$s_{s}=s_{s_{0}}+{Cp}_{s}\ln\frac{T}{T_{0}}$$

where the subscript *s*_0_ represents the reference state, which in this study was assumed to be 303 K, and *Cp_s_* is the specific heat of the salt. The specific heat, enthalpy and entropy of salt at the reference state were 0.8368 kJ/kg·K, 25.104 kJ/kg and 0.08722 kJ/kg·K, respectively.

Brackish water is considered a dilute solution because it has about 4% salinity; thus, it closely approximates the behavior of an ideal solution [22,23].

### 2.3. Economic Assessment

The cost of producing freshwater through desalination depends on several factors, including plant capacity, feedwater quality, pretreatment requirements, the technology employed, energy prices, plant lifetime, and both investment and amortization. The main cost components are the capital investment and the annual operating expenses. The capital cost includes the purchase of the main and auxiliary equipment, installation, and the water pretreatment stage. In contrast, the annual operating costs include all expenses incurred after commissioning and during operation, such as fixed charges or amortization, operation and maintenance (O&M), and membrane replacement [24]. In the present study, the following assumptions were made, according to the methodology described in [11]:

- The expected useful life of the plant (*n*) is 15 years.
- Operation and maintenance costs are estimated at 20% of the annual plant payment.
- The annual membrane replacement rate is 10%.
- The interest rate is 8% when financing is required.
- Effluent water quality: 500 mg/L.

Table 3 and Table 4 present a summary of the main parameters used for cost estimation and the calculated unit cost, respectively.

The amortization factor (*a*) was calculated as follows:

(9) $$a=\frac{{i\left(1+i\right)}^{n}}{{\left(1+i\right)}^{n}-1}$$

where *n* is the plant lifetime and *i* is the interest rate.

## 3. Results

### 3.1. Experimental Performance

**Figure 2:**
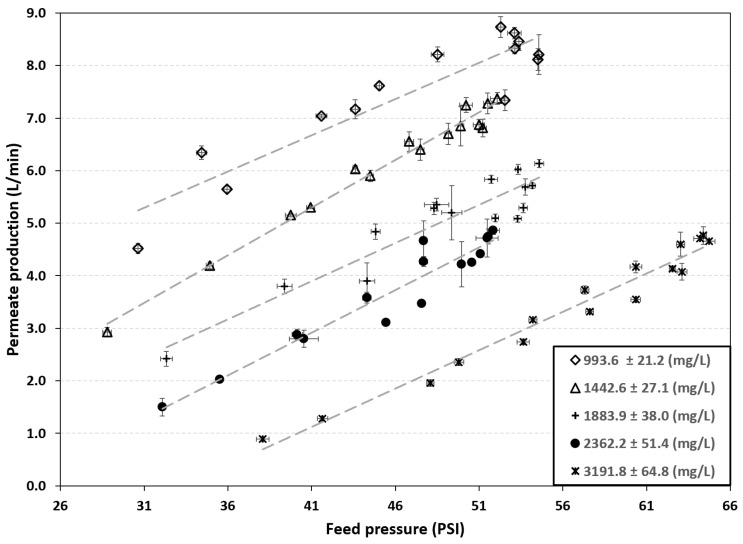
Permeate production at different salt concentrations in the feed stream.

Figure 2 shows the permeate production for different SDT values in the feed. Permeate samples were taken at 30 min intervals. As previously described, the system does not have a battery backup, so its operating pressure depends on the incident solar radiation and varies throughout the day.

According to Figure 2, permeate production increases with increasing feed pressure. However, production decreases as the initial concentration increases. For a feed concentration of 993.6 ± 21.2 mg/L, a maximum of 8.73 L/min a maximum of 8.73 L/min was achieved at a pressure of 53.1 PSI. Furthermore, for an influent with an initial concentration of 3191.8 ± 64.8 mg/L, a maximum production of 4.65 L/min was observed at a pressure of 64.73 PSI.

**Figure 3:**
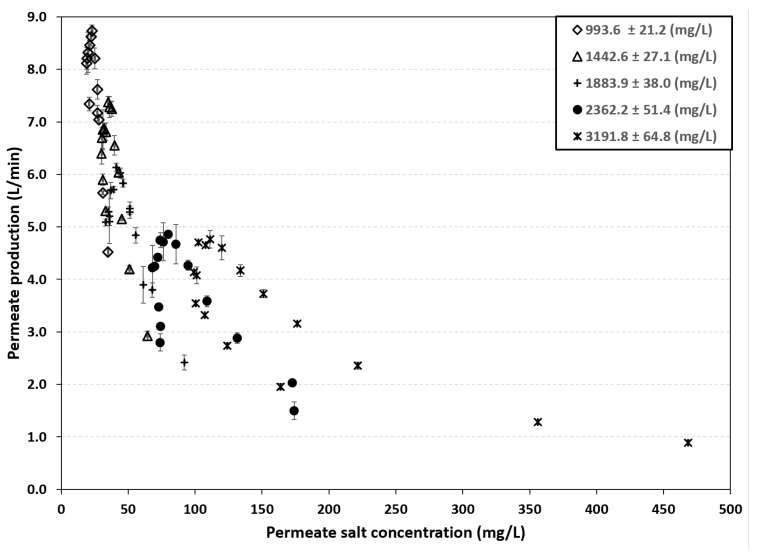
Relationship between permeate salt concentration and production rate.

The data sheet for the RE4040-BLN membrane specifies a salt rejection of 99.20%. This means that a small amount of salt passes through the membrane and affects the final permeate concentration. Figure 3 shows the relationship between permeate concentration and production under five different operating conditions. The figure indicates that as the feed concentration increases, permeate production decreases, while the concentration in the final product increases. For example, for a feed concentration of 993.6 ± 21.2 mg/L and a production rate of 8.73 L/min, the permeate salt concentration was 23 mg/L. In contrast, under operating conditions with a salinity of 3191.8 ± 64.8 mg/L and a production rate of 0.89 L/min, the permeate salt concentration was 468.5 mg/L. This behavior is known as the “dilution effect”: as the operating pressure increases, the permeate flux also increases, while the ion flux remains almost constant, independently of the operating pressure, resulting in lower salt concentration in the produced freshwater [25,26].

The pretreatment stage is used to retain suspended particles and prevent damage to the RO membranes. The gradual accumulation of these particles in the filter causes a pressure drop, which reduces the effective pressure available to drive the separation process. According to the schematic diagram in Figure 1, the pretreatment filter pressure drop is defined as:

(10) $$∆P_{F}=P_{1}-P_{2}$$

**Figure 4:**
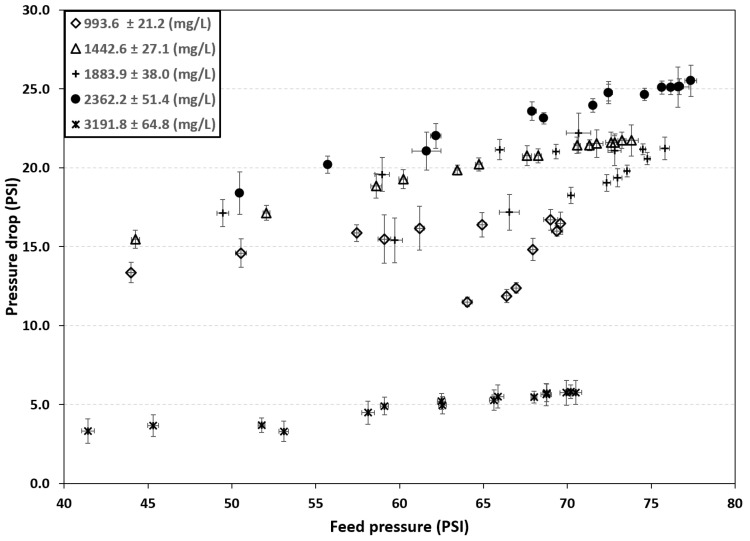
Pretreatment filter pressure drop.

A lower inlet pressure results in a decrease in permeate production. The highest measured pressure drop was 25.5 PSI for a feed pressure of 77.35 psi, whereas under new-filter conditions the pressure drop was 5.76 PSI with a feed pressure of 70.49 PSI (see Figure 4).

The pressure drop produced by the RO membranes was evaluated. According to the schematic diagram in Figure 1, this pressure drop is defined as:

(11) $$∆P_{RO}=P_{2}-P_{3}$$

**Figure 5:**
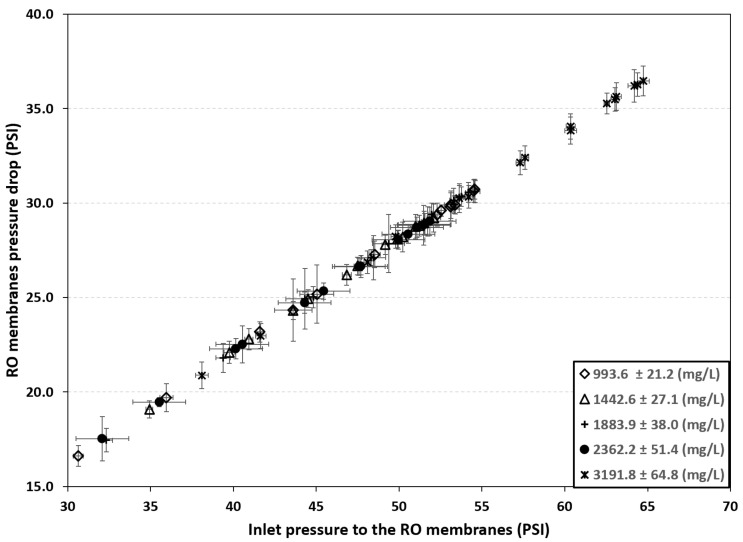
RO membranes pressure drop.

The results are presented in Figure 5. All data for the five operating conditions align along a single trend line, which indicates only a minor fouling effect. However, long-term operating tests are required to observe the fouling behavior of the membranes.

**Figure 6:**
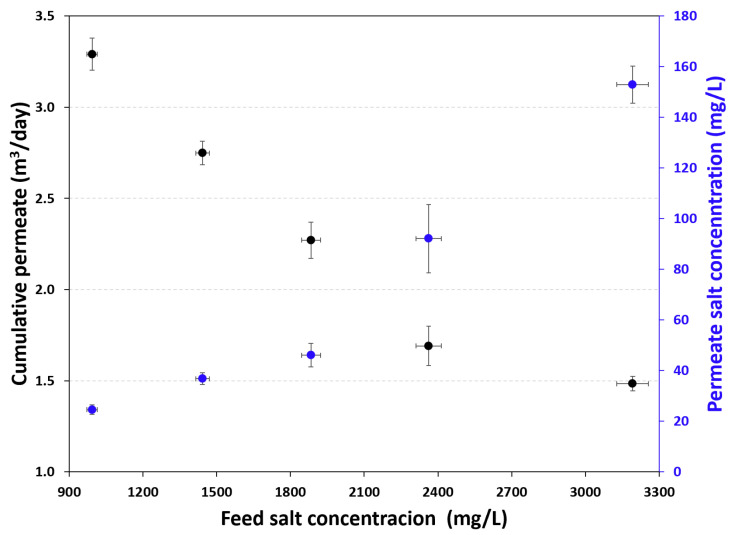
Cumulative permeate production and permeate salt concentration.

The cumulative daily permeate production is shown in Figure 6. This figure indicates that production decreases as the salt concentration of the feed stream increases. However, the total dissolved solids (TDS) concentration in the permeate increases as the salt concentration of the feed stream increases.

### 3.2. Thermodynamic Evaluation

**Figure 7:**
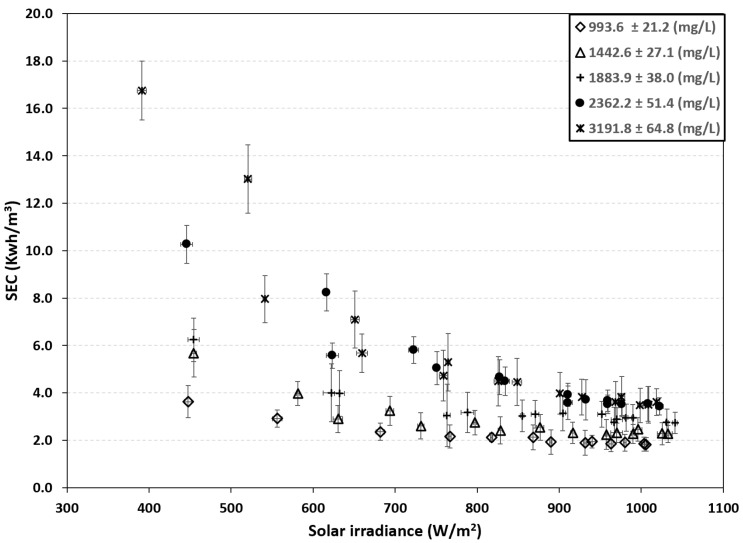
Specific energy consumption (SEC) as a function of solar irradiance.

The decrease in permeate production caused by an increase in feed salt concentration implies a rise in specific energy consumption (see Figure 7). Based on the experimental results, the maximum consumption was 3.61 kWh/m^3^ at a radiation incidence of 1018.47 W/m^2^, while the minimum consumption was 1.80 kWh/m^3^ at a radiation incidence of 1005.99 W/m^2^, corresponding to feed concentrations of 3191.8 ± 64.8 mg/L and 993.6 ± 21.2 mg/L, respectively.

Previous reports show that the energy consumption of PVBWRO systems ranges from 3.5 to 5.0 kWh/m^3^ [27]. The PV-BWRO system without battery support can operate under low radiation conditions (>300 W/m^2^); however, the driving pressure is significantly reduced, which increases the specific energy consumption. The results showed that energy consumption was up to 4.6 times higher than the peak value for an influent concentration of 3191.8 ± 64.8 mg/L when solar irradiance was below 900 W/m^2^.

To evaluate the system’s exergy, the exogenous operating conditions were kept constant over a 1.5 h period at 12:30 to 14:00. A summary of the operating conditions tested is provided in Table 5 and Table 6.

The solar radiation that reaches the PV panels is converted into electricity to drive the pump. The energy efficiency of the solar panels is defined as the ratio between the useful exergy generated by the PV system and the solar input exergy:

(12) $$\eta_{II,PV}=\frac{{Ex}_{PV}}{{Ex}_{sun}}\times \;100$$

The exergy provided by the PV system (*Ex_PV_*) is given by the product of the voltage and current measurement. According to Petela [28], the exergy input to the PV modules (*Ex_sun_*) is:

(13) $${Ex}_{sun}=AI\left[1+\frac{1}{3}{\left(\frac{T_{a}}{T_{sr}}\right)}^{4}-\frac{4}{3}\left(\frac{T_{a}}{T_{sr}}\right)\right]$$

where *T_a_* is the ambient temperature (K), *T_sr_* is the solar radiation temperature (assumed to be 600 K), *A* is the PV module area (m^2^), and *I* is the global irradiation (W/m^2^). During the experiment, the operating conditions were similar; therefore, the exergy efficiency of the PV system was calculated as an average of 11.7%.

The exergy analysis was performed using the stream numbering shown in Figure 1. The figure identifies the critical points at the inlet and outlet of each PV-BWRO system, as well as the discharge streams. Note that points 0, 4, and 6 are evaluated with respect to the reference state.

For the analysis, the exergy flows were determined for each experimental condition. Table 7 summarizes the chemical and physical exergy properties at different points in the system for a representative experimental condition (993.6 ± 21.2 mg/L).

Note that the exergy values at the different points are positive, except for the stream at the reject outlet (point 4). This occurs because the salinity at the reject is higher, and work is required to reduce the concentration to the reference state [21,22,23]. The initial point (0) corresponds to the dead state, so the exergy at this point is also zero.

The exergy entering the system stream is called the input exergy (*Ex_in_*), which is provided by the pump as pressure work and can be expressed as follows.

(14) $${Ex}_{in}={Ex}_{1}-{Ex}_{0}$$

The minimum work input for the desalination process is determined by the chemical properties of the feed stream and the outlet conditions of the system. This balance is evaluated under the reference-state pressure and temperature, considering both the physical and chemical exergy of the stream. According to [21,22], the minimum work can be expressed as follows:

(15) $${\dot{W}}_{min}={Ex}_{6}+{Ex}_{4}-{Ex}_{0}$$

On the other hand, the exergy destroyed is defined as:

(16) $${Ex}_{destroyed}={Ex}_{in}-{\dot{W}}_{min}$$

The second-law efficiency for separation processes was defined as follows: [22]:

(17) $$\eta_{II}=1-\frac{{Ex}_{destroyed}}{{Ex}_{in}}\times \;100$$

The results of Input Exergy, Minimum work, Exergy destroyed and second-law efficiency are shown in Table 8.

The calculated efficiencies were lower than those reported by Cerci [22]. To improve the thermodynamic performance of the PV–BWRO system, it is necessary to identify the components with the greatest exergy loss. The fractions of exergy destroyed in each component are determined as follows:

(18) $$\%{Ex}_{destroyed}=\frac{{Ex}_{destroyed}}{{Ex}_{in}}$$

**Figure 8:**
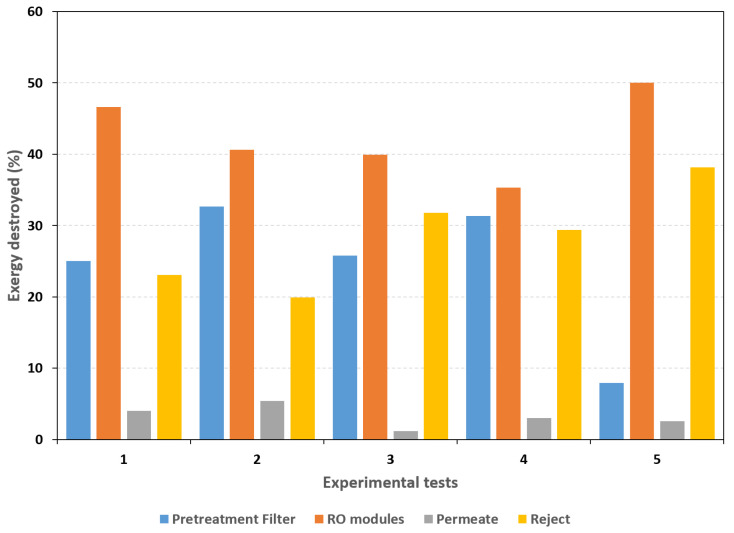
Exergy destruction in each component of the PV-BWRO system.

Figure 8 shows that the greatest exergy loss occurs in the membrane modules, averaging approximately 42.5% of the total exergy input. In tests 1, 2, 3, and 4, the filter generated an average exergy loss of 28.72%, while in the last test it was 7.9%. This difference is due to the pretreatment filter being replaced in this test, as shown in Figure 4, where the pressure drop is lower. The reject stream lost 28.5% of the exergy on average, compared with 3.24% for the permeate. Although the maximum exergy loss occurs in the membrane modules, the combined exergy losses from the reject stream and the filter represent approximately 57.19%, indicating that the system’s low efficiencies are associated with these two components. Regarding the pump, the average exergy efficiency was 38.9%.

To improve system performance, the following strategies are proposed:

(1) Increase the efficiency of the solar panels above 11.7% through maintenance or replacement.
(2) Improve the pretreatment strategy, specifically maintaining continuous operation with an exergy loss near 7.9%.
(3) Recover exergy in the reject stream using a series–parallel configuration.

### 3.3. Cost Analysis

Since the salinity of the permeate is low, subsequent remineralization is required for irrigation applications. For this study, a salinity of 500 mg/L was selected because it is a level that does not affect the yield of many crops [14]. One way to achieve this is by mixing the influent water with permeate to reach a target final concentration. The cost analysis considered the equipment’s technical specifications, design, and daily demineralized production capacity at a target final concentration of 500 mg/L.

**Figure 9:**
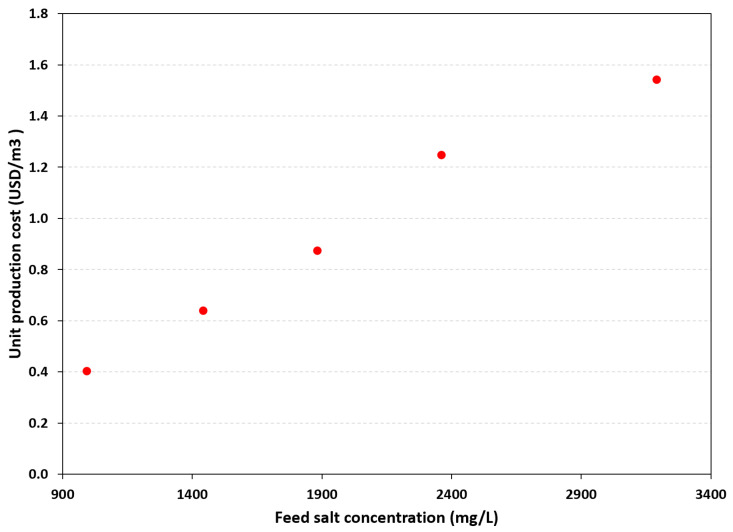
Production cost as a function of feed salt concentration.

The increase in unit production costs is proportional to the influent concentration, as shown in Figure 9. Based on the analysis, a unit production cost of USD 0.49 was obtained for a concentration of 993.6 ± 21.2 mg/L, and a cost of USD 1.87 for a concentration of 3191.8 ± 64.8 mg/L. In both cases, a final permeate TDS concentration of 500 mg/L was considered.

## 4. Conclusions

An experimental evaluation, thermodynamic analysis, and cost estimation of a standalone solar photovoltaic brackish-water reverse osmosis (PV-BWRO) desalination system were conducted. Five salinity conditions were tested, with feed concentrations ranging from 993.6 mg/L to 3191.8 mg/L under open-sky conditions. Based on the experimental results, production decreased as the feed salt concentration increased, dropping from 3.29 m^3^/day (at a salinity of 24.6 mg/L) to 1.48 m^3^/day (at a permeate salinity of 152.9 mg/L). Using a first-law thermodynamic approach, the system’s maximum specific energy consumption was 3.61 kWh/m^3^ at a solar radiation of 1018.47 W/m^2^, while the minimum was 1.80 kWh/m^3^ at 1005.99 W/m^2^.

To identify the components generating the highest entropy and reducing overall effectiveness, an exergy analysis was applied. The solar panel efficiency was 11.7%, while the pump exhibited an efficiency of 38.9%. The average exergy destruction within the system was distributed among the pretreatment stage (28.72%), the membrane modules (42.5%), and the reject stream (28.5%). Although the overall system efficiency was low (maximum of 1.39%), the potential for improvement is significant through maintenance, updated pretreatment strategies, and exergy recovery from the reject stream.

Based on the analysis, a unit production cost of USD 0.49 was obtained for a concentration of 993.6 mg/L, while a cost of USD 1.87 was found for 3191.8 mg/L. These costs were calculated assuming a target final permeate TDS concentration of 500 mg/L.

## Figures and Tables

**Table 1:** Uncertainty of the measured variables.

Variable	Sensor/Instrument	Operation Range	Uncertainty
Temperature	Type T thermocouple	−250 to 350 °C	±0.5 °C
Volumetric flow	Turbine flowmeter	0 to 30 L/min	±1.0 L/min
Pressure	Pressure transducers	0 to 100 psi	±1.0 psi
Solar radiation	Pyranometer	0 to 1000 W/m^2^	±10 W/m^2^
TDS	Multiparameter device	0 to 400 g/L	±1%

**Table 2:** Experimental operating conditions.

Parameter	Value
Salt concentration (mg/L)	993.6 ± 21.2
	1442.6 ± 27.1
	1883.9 ± 38.0
	2362.2 ± 51.4
	3191.8 ± 64.8
Solar Irradiation (W/m^2^) open sky	454.46 to 1041.1

**Table 3:** Main features for cost estimation.

Parameter	Value	Unit
Capital cost (Cc)	6427.8	USD
Membrane module cost (Mc)	1427.8	USD
Capacity (M)	6.5, 4.1, 3.0, 2.1 and 1.7	m^3^/day
Plant availability (f)	90	%
Interest rate	8	%
Operating time	15	year

**Table 4:** Unit cost for freshwater production.

ITEM	Value	Unit
Annual fixed charges	750.95	USD/yr
Membrane replacement	142.78	USD/yr
Operation and maintenance	150.19	USD/yr
Total annual cost	1043.92	USD/yr

**Table 5:** Stream operating conditions.

Variable	Test 1		Test 2		Test 3		Test 4		Test 5	
	FS	Per.	FS	Per.	FS	Per.	FS	Per.	FS	Per.
PH (--)	8.16	8.57	8.34	8.38	8.29	8.08	8.21	7.90	8.19	8.01
TDS (mg/L)	997.94	21.63	1455.19	34.19	1925.25	40.38	2414.25	75.56	3268.31	110.44
Temperature (°C)	33.13	33.72	32.82	34.78	34.52	32.79	34.31	34.88	34.25	34.27
Vol. Flow (L/min)	45.36	8.53	44.81	7.08	47.44	5.89	45.76	4.67	44.75	4.68

FS = Feed stream condition. Per. = Permeate stream condition. Vol. Flow = Volumetric Flow.

**Table 6:** PV system and pressure operating conditions.

	*I* (W/m^2^)	*V* (V)	*C* (A)	P_1_ (PSI)	P_2_ (PSI)	P_3_ (PSI)
Test 1	988.1	146.9	6.5	69.0	53.0	23.2
Test 2	1010.9	153.0	6.4	73.1	51.5	22.5
Test 3	1007.2	153.2	6.5	74.7	54.0	23.6
Test 4	991.5	152.9	6.4	76.7	51.5	22.6
Test 5	998.7	155.6	6.4	69.9	64.1	28.0

I = Solar irradiance. V = Voltage. C = Electric current.

**Table 7:** Exergy properties at different points of the system for 993.6 ± 21.2 mg/L case.

Point	*T* (K)	TDS (mg/L)	*h* (kJ/kg)	*s* (kJ/kg·K)	*ṁ* (kg/min)	*Ψ* (kJ/kg)	Ex (kW)
0	303.0	997.9	125.05	0.436	45.2	0.000	0.000
1	306.1	997.9	138.46	0.479	45.2	0.443	0.334
2	306.1	997.9	138.36	0.479	45.2	0.332	0.250
3	306.1	1234.1	138.16	0.479	36.7	0.046	0.028
4	303.0	1234.1	125.04	0.436	36.7	−0.080	−0.049
5	306.7	21.6	140.64	0.486	8.5	0.470	0.067
6	303.0	21.6	125.11	0.435	8.5	0.375	0.053

**Table 8:** Performance indicators and efficiency for the experimental test.

	Test 1	Test 2	Test 3	Test 4	Test 5
Input Exergy (*Ex_in_*)	0.33359	0.34155	0.43682	0.42237	0.37500
Minimum work (${\dot{W}}_{min}$)	0.00418	0.00474	0.00550	0.00392	0.00503
Exergy destroyed (*Ex_destroyed_*)	0.32941	0.33680	0.43133	0.41845	0.36996
second-law efficiency (η_II_)	1.25%	1.39%	1.26%	0.93%	1.34%

## Data Availability

The data recorders presented in this study are available on request from the corresponding author.
